# Is the Definition of Roma an Important Matter? The Parallel Application of Self and External Classification of Ethnicity in a Population-Based Health Interview Survey

**DOI:** 10.3390/ijerph15020353

**Published:** 2018-02-16

**Authors:** Eszter Anna Janka, Ferenc Vincze, Róza Ádány, János Sándor

**Affiliations:** 1 Department of Dermatology, Faculty of Medicine, University of Debrecen, Debrecen, H-4032, Hungary; janka.eszter.a@gmail.com; 2 Department of Preventive Medicine, Faculty of Public Health, University of Debrecen, Debrecen, H-4012, Hungary; vincze.ferenc@sph.unideb.hu (F.V.); adany.roza@sph.unideb.hu (R.Á.)

**Keywords:** Roma health, ethnicity assessment, self-reporting, observer-reporting, health interview survey

## Abstract

The Roma population is typified by a poor and, due to difficulties in ethnicity assessment, poorly documented health status. We aimed to compare the usefulness of self-reporting and observer-reporting in Roma classification for surveys investigating differences between Roma and non-Roma populations. Both self-reporting and observer-reporting of Roma ethnicity were applied in a population-based health interview survey. A questionnaire was completed by 1849 people aged 18–64 years; this questionnaire provided information on 52 indicators (morbidity, functionality, lifestyle, social capital, accidents, healthcare use) indicators. Multivariate logistic regression models controlling for age, sex, education and employment were used to produce indicators for differences between the self-reported Roma (*N* = 124) and non-Roma (*N* = 1725) populations, as well as between observer-reported Roma (*N* = 179) and non-Roma populations (*N* = 1670). Differences between interviewer-reported and self-reported individuals of Roma ethnicity in statistical inferences were observed for only seven indicators. The self-reporting approach was more sensitive for two indicators, and the observer-reported assessment for five indicators. Based on our results, the self-reported identity can be considered as a useful approach, and the application of observer-reporting cannot considerably increase the usefulness of a survey, because the differences between Roma and non-Roma individuals are much bigger than the differences between indicators produced by self-reported or observer-reported data on individuals of Roma ethnicity.

## 1. Introduction

The Roma population is among the largest minorities in Europe. According to common experiences, which are supported by many data that are not detailed enough to establish effective interventions, their socio-economic and health status is far from acceptable. Despite substantial uncertainties, the EU considers this problem a high priority [1]. This problem also necessitates more systematic research on the role of the Roma ethnicity on health determinants, indicating that the scientific base must be strengthened to establish an adequate Roma policy [2].

Small- and large-scale health surveys are used extensively throughout Europe to assess the population health status. The use of regular surveys to evaluate the health status of the Roma population by inserting the Roma ethnicity into the variables examined during data collection seems to be technically simple and promising. Roma-specific survey results could be very informative. However, this approach is hindered by legal constraints (right to personal data protection of survey participants) and by the poorly elaborated concept of the Roma identity as a social construct [3]. Both practical methods and theoretical strategies require development.

It is widely acknowledged that the effectiveness of surveys involving Roma-specific data collection is limited [1,2]. However, the reliability and, consequently, the usefulness of Roma-specific survey results are not properly characterised. Without improving the methodology for the monitoring of Roma health status, ongoing and future policies have limited effectiveness. The lack of data-driven policy formulation and implementation jeopardises the sustainability of Roma policies in the competitive social environment [4].

Although there is a variability of methods applied in studies to evaluate Roma health status compared to non-Roma populations, the mainstream European approach to identify Roma persons in censuses, surveys and clinical studies is self-reporting. Reports from Bulgaria [5,6,7], Spain [8,9], Slovakia [10,11], Slovenia [12,13], Serbia [14], Belgium [15] and England [16,17,18] apply this approach. Furthermore, this research attitude is also reflected in Hungarian publications on the health status [19,20,21,22] and genetic susceptibility [23,24,25,26] of the Roma people. Due to the fluidity of self-reported identity, which is influenced by societal attitudes, self-reported Roma classification is considered an approximation, which underestimates the proportion of Roma persons and leads to biased results [27]. These limitations are manifested in multi-country studies [28,29,30,31].

Because many people are considered Roma by members of the general population based on external traits, it seems to be a useful approach for interviewers to classify survey participants to prevent biases caused by refused admission of Roma ethnicity in self-reporting [32,33]. According to recent publications, non-self-reported ethnicity can be based on lifestyle [34], surname [35], and residence [36,37,38,39,40], but not on explicitly defined racial characteristics [41,42,43,44,45,46,47]. Health care staff members [48], officers [34], survey interviewers (with or without the support of a Roma community leader [49,50,51,52,53,54,55,56,57,58]), and parents of children [59,60] can perform this classification. Furthermore, the combination of self-reporting and a decisive external classification is also in practice [61,62,63,64,65,66,67,68]. The latter approach clearly shows that external classification is considered more reliable than self-reporting in certain cases. The heterogeneity demonstrates the lack of a standard methodology, as well as the limited comparative value of results from studies with an external Roma classification. Moreover, it is known that the observer-reported identification is influenced by the observer’s ethnicity and sex [27].

The organisation of Roma-specific data collection in different settings to evaluate Roma to non-Roma disparities are hindered by the uncertain nature of the abovementioned classification methods. In reality, the basic choice on self- or external-reporting cannot be based on evidence, because differences between results from interviewer-reporting compared to self-reporting-based surveys are not known in detail. We could not identify any publication in peer reviewed international journals on health interview surveys with a two-fold Roma ethnicity assessment (parallel application of self-reporting and interviewer-reporting) that directly compared the results produced by different Roma definitions.

The objective of our investigation was to assess the health status differences between Roma and non-Roma adults, using both self-reporting and interviewer-reporting for ethnicity for all participants and to describe the differences between the results using the two classification methods. By this comparative study, we aimed to contribute to the debate on the rationality of undertaking this methodological development to handle the legal, ethical, and historical issues related to the external Roma ethnicity assessment.

## 2. Materials and Methods

The survey was implemented in 2015 and covered 20 of 175 Hungarian districts. The list of 3500 persons above 18 years was prepared by randomisation from the population registry of the entire population (*N* = 965,680) with a residential place in the studied districts. Data collection was performed if the subjects signed the informed consent.

The Hungarian adaptation of the European Health Interview Survey [69] was used in the data collection; It was applied in 2009 and 2014 in the Hungarian implementation of the EUROSTAT-organised (European statistics—the statistical office of the European Union) EHIS (European Health Interview Survey). The questionnaire provided information about the general health status, diseases, accidents, functionality, lifestyle, social capital, access to health care, access to preventive services, adherence in drug consumption and oral health. A total of 52 indicators were investigated. Trained interviewers completed the questionnaires. All health indicators were dichotomised before analysis. (Appendix A)

Each respondent’s ethnicity was identified by themselves and by the interviewers. Questions to assess the self-reported ethnicity in the previous Hungarian Census 2011 were added to the survey questionnaire. To counter the low response rate for the ethnicity item, two questions were applied. These questions asked about the ethnicity to which the participant belongs (“Which ethnicity do you feel you belong to?”) and about the other ethnicity to which he or she also belongs (“Do you belong to another ethnicity?”). The primary and secondary self-reported ethnicities were registered in this way. The self-reported Roma category included all interviewees who reported Roma ethnicity either primarily or secondarily.

The interviewers’ Roma classification was part of the questionnaire, which was completed by the interviewers without asking the participants and without informing them about the registered data. The interview was completed at the home of participants. The interviewers’ observations on the visible characteristics and living conditions of the interviewees during the interview formed the bases of categorisation. There were no other specific rules for the interviewers’ Roma identification [65,70]. Similarly to the governmental protocol in the United States, the identification of ethnicity can be carried out by an observer in spite of the acknowledged limitations of external classifications and the practical impossibility of constructing instructions for the observers’ classifications [68].

The questionnaires had been anonymized before entering data into electronic database. The records without personal identifiers had been archived and processed according to the ordinance of the ethical approval.

The socio-demographic determinants of the willingness to declare Roma ethnicity were analysed by multivariate logistic regression. The frequencies of sex, age, education, marital status, employment, and number of persons in the household among participants who self-reported as Roma were compared to those of persons assessed as Roma by only interviewer-based assessment.

The associations between Roma ethnicity and health status indicators were investigated in multivariate logistic regression models applying self-reported and interviewer-reported Roma classifications separately. These models were controlled for age, sex, education, and employment status. The associations were evaluated by the adjusted odds ratios (OR) and their corresponding 95% confidence intervals (95% CI). The results from the two approaches were compared using the 95% confidence intervals by indicators to determine the differences between the two Roma definitions in evaluating the differences between Roma and non-Roma individuals.

Statistical analysis was performed using SPSS 18 (SPSS package for Windows, Release 18; SPSS, Chicago, IL, USA). Being aware of the highly sensitive nature of our investigation from ethical point of view, and preventing ethical restrictions in utilizing results from our investigation, all the ethical regulations had been strictly respected in the whole process of the study. Because the data collection had been implemented in different areas of Hungary, neither an institutional nor a regional ethical committee were competent in evaluating our research plan. Therefore, the detailed study protocol and the applied questionnaire have been reviewed and approved by the highest-level committee of the Medical Research Council, by the Ethical Committee of the Hungarian National Scientific Council on Health (15563-2/2015/EKU 0111/15). 

## 3. Results

The response rate of the survey was 69.2%, with 2421 participants. Because the number of Roma adults was small among the population older than 65 years, the statistical evaluation was restricted to the age range 18–64 years (There were nine self-reported and a further nine interviewer-reported Roma among 572 subjects older than 65.) Ultimately, the investigation focused on 1849 subjects.

**Figure 1:**
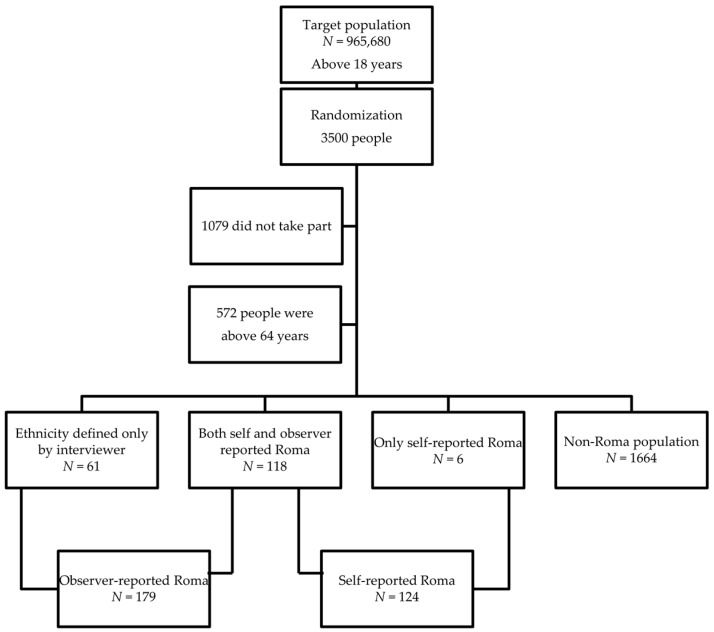
Sampling process of the study.

There were 124 self-reported Roma subjects, whereas 179 people were categorised as Roma ethnicity by interviewers, of whom 61 individuals were identified only by the observers. (Figure 1)

### 3.1. Socio-Economic Status

There was no difference between Roma and non-Roma samples with respect to sex and marital status composition. The Roma age distribution was shifted towards the younger age groups. The economic activity and the level of education were significantly higher among non-Roma individuals. The Roma households were bigger than the non-Roma households. The differences between the Roma and non-Roma were similar, independently of whether ethnicity was assessed via self-report or interviewer-report (Table 1).

According to the multivariate logistic regression analysis, employed Roma individuals were less willing to declare their Roma ethnicity than economically inactive Roma individuals. Similar underreporting of Roma ethnicity was observed in younger age groups with borderline significance (Table 2).

### 3.2. Descriptive Health Status Indicators for Roma

According to the crude descriptive measures, the general health status of the Roma is inferior to that of the non-Roma. There is no difference between Roma and non-Roma individuals with respect to accident frequency and adherence in drug consumption.

Apart from the equal crude prevalence of cardiometabolic disorders, chronic disorders show a higher occurrence among the Roma. Since a higher prevalence is observed for cardiometabolic diseases, the general chronic disease occurrence of the Roma does not deviate significantly from that of the non-Roma. The geographical access to health care is similar among Roma and non-Roma individuals, while the access in terms of time is worse among Roma than among non-Roma individuals. The lifestyle indicators are disadvantageous among the Roma, but two indicators (prevalence of obesity and hearing loss) show no association with the Roma ethnicity.

Almost each functional status and the oral health-related indicators are worse among the Roma. Indicators related to social capital are similar among the Roma and non-Roma. The only exception is that the Roma probably face more difficulties when they need help from neighbours. The ethnic differences in the use of preventive services varies depending on the service.

There are five indicators (difficult to see clearly; not easy to receive help from the neighbours if he/she would need it; cholesterol level was measured in the last year; blood glucose level was measured in the last year; and pulled out teeth because of dental caries or loose teeth) for which the conclusions regarding differences between Roma and non-Roma individuals are not the same when assessing ethnicity by self-reporting vs. interviewer-reporting. Each of the observed differences suggests that the Roma status is worse if the interviewer-reporting approach is applied and is equal to the non-Roma status if the method of self-reported ethnicity is applied (Table 3).

### 3.3. Roma Ethnicity as a Health Determinant Independent of Socio-Economic Status

Using logistic regression to investigate the differences between the characteristics of the two Roma definitions compared to the non-Roma population, it was found that for 33 indicators, there were no remarkable differences, whereas there were significant differences for 14 variables based on both Roma definitions (results for each indicator are presented in detail in Table 4.)

Differences between interviewer-reported and self-reported Roma ethnicity-based ORs were observed for seven indicators. However, the deviations of odds ratios from self-reporting and interviewer-reporting analyses were the same for these seven indicators, and the corresponding confidence intervals showed a wide overlap.

In the self-reporting-based Roma analysis, a body mass index (BMI) above the normal value had less risk of respiratory system disorders (OR: 0.64; 95% CI: 0.41–0.99), whereas respiratory system disorders occurred with higher risk (OR: 1.88; 95% CI: 1.09–3.26) among the Roma. However, the use of glasses or contact lenses (OR: 0.47; 95% CI: 0.28–0.80) and blood glucose measurement in the last year (OR: 0.65; 95% CI: 0.44–0.95) were less likely among the Roma, based on interviewer-reporting analysis. Furthermore, obstructive pain hindering physical activity in the last 4 weeks (OR: 2.23; 95% CI: 1.04–4.79), bleeding gums (OR: 1.87; 95% CI: 1.20–2.90) or lost teeth (OR: 1.85; 95% CI: 1.11–3.08) were more frequent among the Roma in the interviewer-reporting analysis.

**Figure 2:**
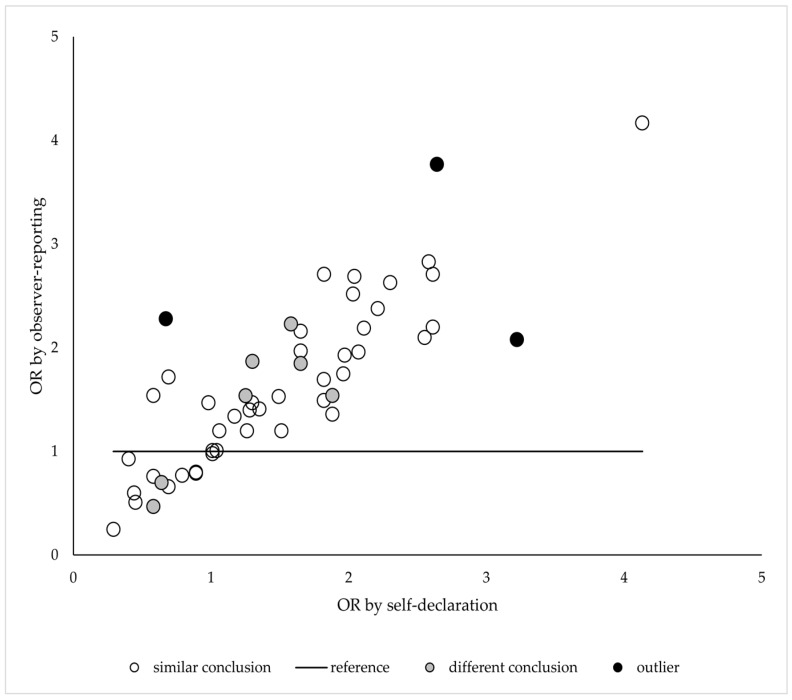
Correlation between socio-economic status adjusted ORs for Roma to non-Roma health status differences from analyses based on self-reported and observer-reported ethnicity assessment by indicators, distinguishing indicators with similar and different statistical conclusions for Roma to non-Roma difference and marking the outlier indicators.

The positive correlation between the point estimates for ORs using the two approaches was strong (*r* = 0.840, *p* < 0.001), with three outliers (risk of road traffic accidents, not taking medicine for respiratory diseases, and tooth cavities without dental filling). Statistical interpretations of the differences between Roma and non-Roma individuals from the two analyses were the same for each outlier (Figure 2).

## 4. Discussion

The self- and external designations of the Roma ethnicity in health surveys were investigated by parallel application to obtain information about the quality of results based on these methodological approaches.

Our observation confirmed the common belief that the observer reports are more effective in identifying Roma adults than the self-reporting approach. In the case of Roma adults, the intention not to admit one’s Roma ethnicity is stronger than the misclassification by an observer who assesses the Roma ethnicity by obtaining information during the interview. In fact, the application of the observer-reported Roma classification resulted in 1.44 times more identified Roma individuals (*N* = 179) than the application of only the self-identification (*N* = 124) approach.

According to the evaluation of the socio-demographic differences between only-observer-identified and self-identified Roma adults, the working Roma are more willing to reject the admission of Roma ethnicity. It is likely that this characteristic is more common among the younger Roma population. Since one of the most important social characteristics of the Roma is their exclusion from the labour market, this profile suggests that the Roma who can break out of this marginalized social position through employment may have a secretive attitude regarding their ethnicity. It seems that this subgroup can be reached by the application of observer reports classifying Roma individuals in health data collection.

The crude descriptive analysis showed significant differences between Roma and non-Roma groups for 35 indicators out of the 52 investigated. There was only one indicator shown to reflect better conditions among the Roma BMI above normal value; ≥25 kg/m^2^). According to the majority of the studied indicators (30 in self-reporting and 35 in observer-reporting analyses), the health status of the Roma was disadvantageous compared to that of the non-Roma. Each difference between self-reporting and observer-reporting results showed the Roma health status as more disadvantageous in the case of observer-reporting. The added value of observer-reporting in Roma health studies can be presented by these 5 out of 52 investigated indicators. This higher effectiveness of the observer-reporting approach in demonstrating health status differences between the Roma and non-Roma can also confirm the lower reliability of the self-reporting of the Roma ethnicity.

Since the Roma ethnicity covaried positively with deprivation, the indicators for Roma-to-non-Roma differences, without adjustment for socio-demographic status, are obviously not informative about the role of Roma ethnicity in influencing risk. The indicators corrected by socio-demographic factors confirmed the results from univariate analyses, such that the Roma health status was shown as inferior to that of the non-Roma (with the exception of BMI above 25 kg/m^2^). However, the number of adjusted indicators with statistically significant Roma-to-non-Roma differences was remarkably reduced in comparison with unadjusted indicators (self-reporting: 15 out of 30; observer-reporting: 15 out of 35). The disadvantageous risk pattern among the Roma is in good concordance with the published results from Hungary [21,65,71,72].

The indicators with statistically significant differences between Roma and non-Roma individuals that could be interpreted differently by self-reporting and observer-reporting do not unequivocally support the higher sensitivity of observer-reporting. Observer-reporting showed higher effectiveness for five of seven indicators, while self-reporting proved to be more effective for two of seven indicators. Our results suggest that the higher effectiveness of observer-reporting in Roma identification, and in crude descriptive evaluation, is not accompanied with higher effectiveness in the evaluation of socio-demographically adjusted indicators.

Our results show that it is causeless to undertake the elaboration of methodology that can handle all the sensitive (historical, legal, and ethical) issues related to the external Roma classification. There is a low probability that the survey results based on external Roma classification could improve the effectiveness of data-driven health policy formulation.

### Strengths and Limitations

The present study was a population-based investigation with the sample selected at random. The size of the non-Roma population was considerably large, ensuring relatively precise reference values for Roma-specific risk evaluation. The quality of collected data was ensured by the application of questions from the European Health Interview Survey, which was tested in a Hungarian national survey as well. The health-determining role of ethnicity could be studied with control for deprivation because Roma-specific risks were adjusted for a number of socio-demographic factors. The main strength of this study was the parallel use of self-reporting and interview-reporting identification, allowing a direct comparison of the two methods.

The most important limitations of our study were the low response rate and the weak statistical power because of the relatively small number of Roma subjects in the studied sample. This small number of Roma subjects likely resulted in a type II error, which is responsible for the lack of any observable differences between the Roma risks computed by the two approaches, whereas many Roma-to-non-Roma differences were detected by both methods.

We could not investigate the added value of interviewer-reporting ethnicity assessment as extra question in survey added to questions on the self-reported ethnicity. Odds ratios for interaction could not be computed by logistic regression models with term for interaction between self-reported and interviewer-reported Roma ethnicity in case of many indicators, because of the small number of Roma participants in our survey (data not shown). Therefore, the direct measure for the added value of interviewer-reporting ethnicity assessment as additional question could not been computed using our database. On the other hand, according to the logistic regression models which distinguished (a) the Roma by self-reporting irrespective of the result of interviewer-reporting; (b) the interviewer-reported Roma without admitted Roma ethnicity; (c) non-Roma classified by both self-reporting and interviewer reporting, there was no indicator with significant difference between the two Roma groups. Due to the small number of Roma participants, the lack of significant difference was accompanied with wide 95% confidence intervals (Appendix B).

Our study investigated one important source of uncertainty of Roma health studies. We could not investigate the role of the interaction between the observer’s and interviewee’s personality and the interview conditions. Furthermore, we could not investigate how uncertainties of the social construct for the Roma ethnicity can influence the ethnicity classification.

## 5. Conclusions

Although the young and employed Roma seem to be less willing to declare their Roma ethnicity than the older and unemployed Roma, there is no remarkable discrepancy in survey conclusions in the difference between Roma and non-Roma adults’ health status if we use ethnicity data based on self-reporting or interviewer-reporting. Based on our observations adjusted by socio-demographic status, both approaches for ethnicity identification are equally applicable in surveys, and it seems that the hesitation to insert self-reported Roma ethnicity into the set of surveyed indicators due to the assumed uncertain nature of self-identification is not justified.

The health status differences between the Roma and non-Roma are much larger than those between self-reported and interviewer-reported Roma. Therefore, the issues related to the value of self-reported Roma ethnicity data are not reasonable to prevent extending these surveys by Roma-specific data collection, despite the fact that the Roma identification based on the combination of self-reporting and interviewer-reporting approaches yields remarkably larger Roma subgroups in surveys.

## Figures and Tables

**Table 1:** Socio-economic status of the Roma and non-Roma adults.

Category	Variables	Self-Reported Roma (*N* = 124)	Non-Roma by Self-Reporting (*N* = 1725)	*p*-Value ^+^	Ethnicity Defined by the Interviewer (*N* = 179)	Non-Roma Population by Interviewer-Reporting (*N* = 1670)	*p*-Value ^+^
Sex	Male	45.97% (57)	49.28% (850)	0.477	44.13% (79)	49.58% (828)	0.166
	Female	54.03% (67)	50.72% (875)		55.87% (100)	50.42% (842)	
Age	18–24	22.58% (28)	13.04% (225)	0.006 *	24.02% (43)	12.57% (210)	<0.001 *
	25–34	23.39% (29)	18.61% (321)		22.91% (41)	18.50% (309)	
	35–44	20.16% (25)	24.75% (427)		22.35% (40)	24.67% (412)	
	45–54	20.16% (25)	20.58% (355)		20.67% (37)	20.54% (343)	
	55–64	13.71% (17)	23.01% (397)		10.06% (18)	23.71% (396)	
Education	Higher than primary school	29.84% (37)	87.30% (1506)	<0.001 *	31.84% (57)	88.98% (1486)	<0.001 *
	Primary school or lower education	70.16% (87)	12.70% (219)		68.16% (122)	11.02% (184)	
Marital status	Married	59.68% (74)	55.00% (940)	0.312	56.50% (100)	55.19% (914)	0.740
	Single-divorced-widowed-separated	40.32% (50)	45.00% (769)		43.50% (77)	44.81% (742)	
Economic activity	Full-time employee + part-time employee + temporary employee	42.74% (53)	71.30% (1227)	<0.001 *	48.04% (86)	71.67% (1194)	<0.001 *
	Other inactive + retired + student + cared	37.10% (46)	22.78% (392)		35.20% (63)	22.51% (375)	
	Unemployed	20.16% (25)	5.93% (102)		16.76% (30)	5.82% (97)	
The number of persons in a household	Lives alone	7.26% (9)	15.01% (259)	<0.001 *	6.15% (11)	15.39% (257)	<0.001 *
	Two-person	18.55% (23)	32.41% (559)		19.55% (35)	32.75% (547)	
	Three-person	20.97% (26)	24.75% (427)		21.23% (38)	24.85% (415)	
	Four-person	15.32% (19)	18.49% (319)		18.44% (33)	18.26% (305)	
	Five-person	20.16% (25)	6.26% (108)		19.55% (35)	5.87% (98)	
	Six or more	17.74% (22)	3.07% (53)		15.08% (27)	2.87% (48)	

^+^ χ^2^ test; * Significant results (*p* < 0.05).

**Table 2:** Socio-economic status of only-interviewer-reported Roma adults compared to the self-reported Roma according to multivariate logistic regression.

	OR (95% CI) ^+^
Sex	
female/male	1.26 (0.62; 2.57)
Age (years old)	
18–24/55–64	5.08 (0.98; 26.49) *
25–34/55–64	3.19 (0.63; 16.18)
35–44/55–64	5.06 (1.00; 25.54) *
45–54/55–64	3.45 (0.74; 16.01)
Education	
(higher than primary school)/(primary school or lower education)	1.01 (0.48; 2.12)
Marital status	
married/(single-divorced-widowed-separated)	0.79 (0.38; 1.62)
Economic activity	
(full-time employee + part-time employee + temporary employee)/unemployed	3.49 (1.17; 10.41) *
(other inactive + retired + student + cared)/unemployed	2.70 (0.86; 8.46)
The number of persons in a household	
two-person/lives alone	3.25 (0.51; 20.62)
three-person/lives alone	1.79 (0.28; 11.43)
four-person/lives alone	2.62 (0.39; 17.82)
five-person/lives alone	1.41 (0.21; 9.64)
six or more/lives alone	0.95 (0.12; 7.47)

^+^ OR (95% CI): Odds ratios (95% Confidence intervals). * Significant results.

**Table 3:** Unadjusted descriptive health status indicators for the Roma and non-Roma adults.

Categories	Indicators	Self-Reported Roma Classification			Interviewer-Reported Roma Classification		
		Roma (*N* = 124)	Non-Roma (*N* = 1725)	*p*-Value ^+^	Roma (*N* = 179)	Non-Roma (*N* = 1670)	*p*-Value ^+^
General health status	Health status is satisfactory or worse (vs. good, very good)	47.58% (59)	25.86% (446)	<0.001 *	44.13% (79)	25.51% (426)	<0.001 *
	He/she can do little for his/her health	50.00% (60)	21.55% (367)	<0.001 *	46.55% (81)	20.98% (346)	<0.001 *
	He/she find that his/her teeth are in bad condition	35.77% (44)	13.74% (236)	<0.001 *	34.83% (62)	13.12% (218)	<0.001 *
Diseases	Chronic disease, which exists for 6 months	30.33% (37)	26.00% (448)	0.294	27.12% (48)	26.20% (437)	0.792
	Musculoskeletal disorders	28.23% (35)	14.84% (256)	<0.001 *	23.46% (42)	14.91% (249)	0.003 *
	Cardiometabolic diseases	29.84% (37)	24.46% (422)	0.181	27.37% (49)	24.55% (410)	0.406
	Digestive disorders and excretory system diseases	10.48% (13)	3.36% (58)	<0.001 *	8.94% (16)	3.29% (55)	<0.001 *
	Mental disorders	10.48% (13)	3.36% (58)	<0.001 *	7.82% (14)	3.41% (57)	0.004 *
	Respiratory system disorders (allergic diseases also)	18.55% (23)	10.49% (181)	0.006 *	15.64% (28)	10.54% (176)	0.038 *
Accidents	Road traffic accident	2.42% (3)	1.28% (22)	0.287	1.68% (3)	1.32% (22)	0.694
	Home accident	2.42% (3)	4.64% (80)	0.249	5.03% (9)	4.43% (74)	0.715
	Leisure activity accident	0.81% (1)	2.38% (41)	0.256	1.12% (2)	2.4% (40)	0.275
Functionality	Health problem obstructs him/her in the last 6 months	32.26% (40)	18.02% (310)	<0.001 *	26.82% (48)	18.14% (302)	0.005 *
	In the past 4 weeks had physical pain	51.61% (64)	31.53% (541)	<0.001 *	50.56% (90)	30.99% (515)	<0.001 *
	In the past 4 weeks, physical pain has hindered his/her activities	85.94% (55)	70.11% (380)	0.008 *	86.67% (78)	69.19% (357)	0.001 *
	Difficult to see sharply with glasses	44.44% (8)	17.01% (92)	0.003 *	40.91% (9)	16.95% (91)	0.004 *
	Difficult to see clearly	6.60% (7)	6.35% (75)	0.917	10.19% (16)	5.84% (66)	0.036 *
	Use of glasses or contact lenses	14.52% (18)	31.44% (541)	<0.001 *	12.29% (22)	32.23% (537)	<0.001 *
	Difficult to hear well in a noisy room	6.67% (8)	6.00% (97)	0.767	5.81% (10)	6.07% (95)	0.893
	Difficult to walk 500 m on flat ground without help	15.32% (19)	7.80% (134)	0.003 *	13.41% (24)	7.76% (129)	0.009 *
	Difficult to descend or climb 12 steps	16.94% (21)	10.02% (172)	0.015 *	15.08% (27)	9.99% (166)	0.035 *
Lifestyle	BMI above normal value (≥25 kg/m^2^)	41.13% (51)	52.29% (902)	0.016 *	41.90% (75)	52.57% (878)	0.007 *
	Obesity (BMI ≥ 30 kg/m^2^)	12.10% (15)	15.54% (268)	0.304	13.97% (25)	15.45% (258)	0.601
	More active, more labour-intensive work	92.00% (92)	67.44% (1046)	<0.001 *	91.22% (135)	66.73% (1003)	<0.001 *
	Never do sports	91.06% (112)	74.97% (1282)	<0.001 *	90.45% (161)	74.50% (1233)	<0.001 *
	Fruits consumption maximum 1–3 times per week	56.45% (70)	30.48% (524)	<0.001 *	55.31% (99)	29.75% (495)	<0.001 *
	Vegetables consumption maximum 1–3 times per week	58.87% (73)	39.06% (671)	<0.001 *	55.31% (99)	38.79% (645)	<0.001 *
	Currently smoking	58.87% (73)	31.36% (539)	<0.001 *	61.45% (110)	30.17% (502)	<0.001 *
	Minimum 2 to 3 times a month drinks 6 or more drinks containing alcohol	4.76% (2)	9.20% (63)	0.328	8.47% (5)	8.98% (60)	0.896
Social capital	Can expect to help maximum 2 people in case of personal problems	35.48% (44)	38.03% (653)	0.572	34.64% (62)	38.21% (635)	0.349
	Others do not show much interest to him/her	72.95% (89)	72.40% (1225)	0.895	72.47% (129)	72.43% (1185)	0.991
	Not easy to receive help from the neighbours if he/she would need it	39.02% (48)	30.56% (510)	0.050	39.43% (69)	30.24% (489)	0.013*
	He/she cannot talk to anyone about his/her personal cases	4.03% (5)	3.49% (60)	0.754	3.91% (7)	3.49% (58)	0.772
Access to health care	Late medical care because of waiting	25.00% (12)	11.29% (57)	0.006 *	22.73% (15)	11.09% (54)	0.007 *
	Late medical care because of long distance	10.42% (5)	4.74% (24)	0.092	9.09% (6)	4.71% (23)	0.134
Access to preventive services	**This year or last year got flu vaccine**	**50.00% (6)**	**34.11% (73)**	**0.261**	**46.67% (7)**	**34.12% (72)**	**0.325**
	Cholesterol level was measured in the last year	34.17% (41)	42.09% (705)	0.089	31.98% (55)	42.58% (691)	0.007 *
	Blood glucose level was measured in the last year	38.52% (47)	44.99% (759)	0.165	35.03% (62)	45.59% (744)	0.007 *
	Mammography examination in the last 2 years	18.75% (12)	38.54% (333)	0.002 *	20.83% (20)	39.06% (325)	<0.001 *
	Cytological examination in the last 3 years	39.68% (25)	69.57% (599)	<0.001 *	43.16% (41)	70.33% (583)	<0.001 *
Adherence in drug consumption	People who take no medicines in case of musculoskeletal disorders	20.00% (7)	24.61% (63)	0.550	21.43% (9)	24.50% (61)	0.667
	People who take no medicines in case of cardiometabolic diseases	10.81% (4)	9.48% (40)	0.792	12.24% (6)	9.27% (38)	0.504
	People who take no medicines in case of digestive and excretory system diseases	7.69% (1)	12.07% (7)	0.652	6.25% (1)	12.73% (7)	0.471
	People who take no medicines in case of respiratory system disorders (allergic diseases also)	17.39% (4)	18.78% (34)	0.872	28.57% (8)	17.05% (30)	0.146
Oral health	Carious tooth/cavity	58.06% (72)	27.18% (461)	<0.001 *	61.45% (110)	25.78% (423)	<0.001 *
	Dental fillings	50.81% (63)	78.53% (1342)	<0.001 *	55.87% (100)	78.9% (1305)	<0.001 *
	Bleeding gums when tooth brushing	21.77% (27)	13.93% (237)	0.017 *	24.58% (44)	13.37% (220)	<0.001 *
	Lost teeth	20.16% (25)	8.70% (148)	<0.001 *	18.99% (34)	8.44% (139)	<0.001 *
	Pulled out teeth because of dental caries or loose teeth	66.13% (82)	57.46% (978)	0.059	69.83% (125)	56.77% (935)	<0.001 *
	Prosthesis or other type of dentures	16.13% (20)	31.97% (548)	<0.001 *	15.08% (27)	32.61% (541)	<0.001 *
	Missing teeth without prosthesis	69.35% (86)	46.04% (784)	<0.001 *	70.95% (127)	45.08% (743)	<0.001 *
	No dental filling, but he/she has cavity	25.00% (31)	4.93% (85)	<0.001 *	24.58% (44)	4.31% (72)	<0.001 *

^+^ χ^2^ test; * Significant results (*p* < 0.05). BMI: Body Mass Index.

**Table 4:** Health determining role of Roma ethnicity according self-reported and interviewer-reported Roma ethnicity assessment (odds ratios with 95% confidence intervals in parentheses from multivariate logistic regression models controlled for age, sex, education and employment).

Categories	Indicators	Self-Reported Roma Ethnicity	Interviewer-Reported Roma Ethnicity
General health status	Health status is satisfactory or worse (vs. good, very good)	2.11 (1.28; 3.49)) *	2.19 (1.40; 3.42) *
	He/she can do little for his/her health	2.61 (1.68; 4.06) *	2.71 (1.84; 4.01) *
	He/she find that his/her teeth are in bad condition	2.03 (1.25; 3.29) *	2.52 (1.62; 3.90) *
Diseases	Chronic disease, which exists for 6 months	0.89 (0.54; 1.47)	0.79 (0.50; 1.23)
	Musculoskeletal disorders	2.55 (1.51; 4.31) *	2.10 (1.30; 3.40) *
	Cardio-metabolic diseases	1.04 (0.62; 1.77)	1.01 (0.63; 1.62)
	Digestive disorders and excretory system diseases	2.07 (0.98; 4.38)	1.96 (0.96; 4.02)
	Mental disorders	1.88 (0.87; 4.07)	1.36 (0.64; 2.89)
	Respiratory system disorders (allergic diseases also)	1.88 (1.09; 3.26) *	1.54 (0.92; 2.58)
Accidents	Road traffic accident	3.22 (0.75; 13.86)	2.08 (0.49; 8.83)
	Home accident	0.58 (0.17; 1.98)	1.54 (0.68; 3.47)
	Leisure activity accident	0.44 (0.06; 3.57)	0.60 (0.13; 2.85)
Functionality	Health problem obstructs him/her in the last 6 months	1.51 (0.92; 2.49)	1.20 (0.76; 1.89)
	In the past 4 weeks had physical pain	2.30 (1.48; 3.58) *	2.63 (1.78; 3.88) *
	In the past 4 weeks, physical pain has hindered his/her activities	1.58 (0.69; 3.61)	2.23 (1.04; 4.79) *
	Difficult to see sharply with glasses	1.97 (0.69; 5.58)	1.93 (0.72; 5.16)
	Difficult to see clearly	0.69 (0.28; 1.74)	1.72 (0.83; 3.56)
	Use of glasses or contact lenses	0.58 (0.32; 1.03)	0.47 (0.28; 0.80) *
	Difficult to hear well in a noisy room	1.26 (0.54; 2.90)	1.20 (0.55; 2.60)
	Difficult to walk 500 m on flat ground without help	1.49 (0.78; 2.84)	1.53 (0.84; 2.79)
	Difficult to descend or climb 12 steps	1.35 (0.73; 2.51)	1.41 (0.80; 2.50)
Lifestyle	BMI above normal value (≥25 kg/m^2^)	0.64 (0.41; 0.99) *	0.70 (0.48; 1.03)
	Obesity (BMI ≥ 30 kg/m^2^)	0.58 (0.32; 1.07)	0.76 (0.45; 1.26)
	More active, more labour-intensive work	4.13 (1.94; 8.81) *	4.17 (2.26; 7.70) *
	Never do sports	2.58 (1.29; 5.17) *	2.83 (1.58; 5.06) *
	Fruits consumption maximum 1–3 times per week	2.21 (1.46; 3.34) *	2.38 (1.65; 3.42) *
	Vegetables consumption maximum 1–3 times per week	1.96 (1.30; 2.95) *	1.75 (1.22; 2.49) *
	Currently smoking	2.04 (1.34; 3.12) *	2.69 (1.85; 3.91) *
	Drinks 6 or more drinks containing alcohol a minimum 2 to 3 times a month	0.40 (0.09; 1.91)	0.93 (0.32; 2.73)
Social capital	Can expect to help maximum 2 people in case of personal problems	0.79 (0.52; 1.21)	0.77 (0.53; 1.13)
	Others do not show much interest to him/her	1.01(0.64; 1.59)	0.98 (0.66; 1.47)
	Not easy to receive help from the neighbours if he/she would need it	1.28 (0.84; 1.94)	1.40 (0.96; 2.03)
	He/she cannot talk to anyone about his/her personal cases	1.01 (0.36; 2.83)	1.01 (0.40; 2.52)
Access to health care	Late medical care because of waiting	1.65 (0.69; 3.95)	1.97 (0.87; 4.50)
	Late medical care because of long distance	1.17 (0.34; 4.00)	1.34 (0.42; 4.26)
Access to preventive services	This year or last year got flu vaccine	2.61 (0.63; 10.94)	2.20 (0.54; 8.94)
	Cholesterol level was measured in the last year	0.77 (0.49; 1.20)	0.68 (0.46; 1.02)
	Blood glucose level was measured in the last year	0.80 (0.52; 1.24)	0.65 (0.44; 0.95) *
	Mammography examination in the last 2 years	0.55 (0.27; 1.13)	0.67 (0.37; 1.21)
	Cytological examination in the last 3 years	0.55 (0.30; 1.01)	0.59 (0.35; 1.01)
Adherence in drug consumption	People who take no medicines for musculoskeletal disorders	0.89 (0.32; 2.43)	0.80 (0.30; 2.14)
	People who take no medicines for cardiometabolic diseases	1.06 (0.30; 3.71)	1.20 (0.39; 3.71)
	People who take no medicines in case of digestive and excretory system diseases	0.29 (0.02; 4.62)	0.25 (0.02; 3.90)
	People who take no medicines in case of respiratory system disorders (allergic diseases also)	0.67 (0.16; 2.87)	2.28 (0.65; 8.06)
Oral health	Carious tooth/cavity	1.82 (1.18; 2.80) *	2.71 (1.86; 3.95) *
	Dental fillings	0.45 (0.29; 0.68) *	0.51 (0.35; 0.74) *
	Bleeding gums when tooth brushing	1.30 (0.78; 2.16)	1.87 (1.20; 2.90) *
	Lost teeth	1.65 (0.95; 2.87)	1.85 (1.11; 3.08) *
	Pulled out teeth because of dental caries or loose teeth	0.98 (0.61; 1.56)	1.47 (0.98; 2.23)
	Prosthesis or other type of dentures	0.69 (0.39; 1.22)	0.66 (0.40; 1.09)
	Missing teeth without prosthesis	1.65 (1.05; 2.60) *	2.16 (1.45; 3.21) *
	No dental filling, but he/she has cavity	2.64 (1.52; 4.58) *	(2.23; 6.39) *

* Significant results.

## Appendix A

The list of indicators with the dichotomized categories based on the aggregation of original answers collected according to the European Health Interview Survey questions.

### Appendix A.1. General Health Status

1. Health status:
  - satisfactory or worse
  - good, very good
2. Thinking about how much can do for his/her health
  - he/she can do little for his/her health
  - he/she can do much for his/her health
3. Teeth condition
  - he/she find that his/her teeth are in bad condition
  - he/she find that his/her teeth are in good condition

### Appendix A.2. Disease

1. Chronic disease, which exists for 6 months
  - yes
  - no
2. Musculoskeletal disorders
  - yes
  - no
3. Cardiometabolic diseases
  - yes
  - no
4. Digestive disorders and excretory system diseases
  - yes
  - no
5. Mental disorders
  - yes
  - no
6. Respiratory system disorders (allergic diseases also)
  - yes
  - no

### Appendix A.3. Accidents

1. Road traffic accident
  - yes
  - no
2. Home accident
  - yes
  - no
3. Leisure activity accident
  - yes
  - no

### Appendix A.4. Functionality

1. Health problem obstructs him/her in the last 6 months
  - yes
  - no
2. In the past 4 weeks had a physical pain
  - yes
  - no
3. In the past 4 weeks, physical pain has hindered his/her activities
  - yes
  - no
4. Difficult to see sharply with glasses
  - yes
  - no
5. Difficult to see clearly
  - yes
  - no
6. Use of glasses or contact lenses
  - yes
  - no
7. Difficult to hear well in a noisy room
  - yes
  - no
8. Difficult to walk 500 m on flat ground without help
  - yes
  - no
9. Difficult to descend or climb 12 steps
  - yes
  - no

### Appendix A.5. Lifestyle

1. BMI above normal value (≥25 kg/m^2^)
  - BMI ≥ 25 kg/m^2^
  - BMI < 25 kg/m^2^
2. Obesity (BMI ≥ 30 kg/m^2^)
  - BMI ≥ 30 kg/m^2^
  - BMI < 30 kg/m^2^
3. Work activity
  - more active, more labour-intensive work
  - passive work
4. Sport
  - never do sports
  - do sports
5. Fruit consumption
  - maximum 1–3 times per week
  - minimum 4–6 times per week
6. Vegetable consumption
  - maximum 1–3 times per week
  - minimum 4–6 times per week
7. Smoking
  - currently
  - never smoked or stopped
8. Alcohol drinking
  - minimum 2 to 3 times a month drinks 6 or more drinks containing alcohol
  - maximum 1 time a month drinks 6 or more drinks containing alcohol

### Appendix A.6. Social Capital

1. In case of personal problems, how many people can expect to help
  - can expect maximum 2 people in case of personal problem
  - can expect more people in case of personal problem
2. Other people’s interest to him/her
  - others do not show much interest to him/her
  - others show much interest to him/her
3. Receive help from the neighbours if he/she would need it
  - not easy to receive help from the neighbours if he/she would need it
  - easy to receive help from the neighbours if he/she would need it
4. He/she can talk to anyone about his/her personal cases
  - no
  - yes

### Appendix A.7. Access to Health Care

1. Late medical care received because of waiting
  - yes
  - no
2. Late medical care received because of long distance
  - yes
  - no

### Appendix A.8. Access to Preventive Services

1. Flu vaccine
  - this year or last year
  - more than one year
2. Cholesterol level
  - cholesterol level was measured in the last year
  - cholesterol level was measured for more than one year
3. Blood glucose level
  - blood glucose level was measured in the last year
  - blood glucose level was measured for more than one year
4. Mammography examination
  - in the last 2 years
  - for more than 2 years
5. Cytological examination
  - in the last 3 years
  - for more than 3 years

### Appendix A.9. Adherence in Drug Consumption

1. People who take medicines in case of musculoskeletal disorders
  - no
  - yes
2. People who take medicines in case of cardiometabolic diseases
  - no
  - yes
3. People who take medicines in case of digestive and excretory system diseases
  - no
  - yes
4. People who take medicines in case of respiratory system disorders (allergic diseases also)
  - no
  - yes

### Appendix A.10. Oral Health

1. Carious tooth/cavity
  - yes
  - no
2. Dental fillings
  - yes
  - no
3. Bleeding gums when tooth brushing
  - yes
  - no
4. Lost teeth
  - yes
  - no
5. Pulled out teeth because of dental caries or loose teeth
  - yes
  - no
6. Prosthesis or other type of dentures
  - yes
  - no
7. Missing teeth without prosthesis
  - yes
  - no
8. No dental filling, but he/she has cavity
  - yes
  - no

## Appendix B

**Table A1:** Health risks among the Roma compared to non-Roma (according to both self and interviewer ethnicity assessment) by self-reported and by ONLY the interviewer-reported Roma ethnicity (odds ratios with 95% confidence intervals in square brackets from one multivariate logistic regression model controlled for age, sex, education and employment).

	Indicators	Both Self and External Identification of Roma Ethnicity AND Only Self-Reported Roma *N* = 124 vs. Non-Roma Population *N* = 1664	Only Interview-Reported Roma *N* = 61 vs. Non-Roma Population *N* = 1664
General health status	Health status is satisfactory or worse (vs. good, very good)	2.42 (1.45; 4.04) *	2.41 (1.25; 4.65) *
	He/she can do little for his/her health	3.00 (1.92; 4.69) *	2.67 (1.48; 4.82) *
	He/she find that his/her teeth are in bad condition	2.43 (1.48; 3.99) *	3.04 (1.59; 5.81) *
Diseases	Chronic disease, which exists for 6 months	0.87 (0.53; 1.45)	0.85 (0.43; 1.70)
	Musculoskeletal disorders	2.63 (1.54; 4.48) *	1.33 (0.59; 2.98)
	Cardio-metabolic diseases	1.04 (0.61; 1.77)	0.95 (0.46; 1.99)
	Digestive disorders and excretory system diseases	2.11 (0.98; 4.55)	1.14 (0.32; 4.11)
	Mental disorders	1.81 (0.82; 3.97)	0.69 (0.15; 3.20)
	Respiratory system disorders (allergic diseases also)	1.82 (1.04; 3.19) *	0.76 (0.29; 2.00)
Accidents	Road traffic accident	2.98 (0.68; 13.06)	nc
	Home accident	0.70 (0.20; 2.43)	3.15 (1.20; 8.30) *
	Leisure activity accident	0.43 (0.05; 3.55)	0.83 (0.10; 6.82)
Functionality	Health problem obstructs him/her in the last 6 months	1.54 (0.93; 2.55)	1.11 (0.54; 2.31)
	In the past 4 weeks had a physical pain	2.64 (1.68; 4.14) *	2.63 (1.46; 4.74) *
	In the past 4 weeks, physical pain has hindered his/her activities	1.87 (0.80; 4.34)	3.68 (0.97; 13.97)
	Difficult to see sharply with glasses	2.05 (0.72; 5.82)	3.16 (0.46; 21.61)
	Difficult to see clearly	0.91 (0.35; 2.34)	3.30 (1.34; 8.08) *
	Use of glasses or contact lenses	0.53 (0.30; 0.95) *	0.35 (0.13; 0.92) *
	Difficult to hear well in a noisy room	1.23 (0.53; 2.86)	0.78 (0.18; 3.47)
	Difficult to walk 500 m on flat ground without help	1.60 (0.83; 3.08)	1.74 (0.67; 4.52)
	Difficult to descend or climb 12 steps	1.43 (0.76; 2.69)	1.62 (0.66; 4.00)
Lifestyle	BMI above normal value (≥25 kg/m^2^)	0.63 (0.40; 0.98) *	0.86 (0.48; 1.54)
	Obesity (BMI ≥ 30 kg/m^2^)	0.58 (0.31; 1.08)	1.01 (0.48; 2.14)
	More active, more labour-intensive work	4.54 (2.12; 9.73) *	3.11 (1.28; 7.53)*
	Never do sports	2.85 (1.41; 5.75) *	2.53 (1.06; 6.03) *
	Fruits consumption maximum 1–3 times per week	2.46 (1.61; 3.74) *	2.18 (1.25; 3.78) *
	Vegetables consumption maximum 1–3 times per week	2.07 (1.37; 3.13) *	1.50 (0.87; 2.58)
	Currently smoking	2.37 (1.54; 3.64) *	3.02 (1.70; 5.38) *
	Minimum 2 to 3 times a month drinks 6 or more drinks containing alcohol	0.44 (0.09; 2.08)	1.80 (0.47; 6.88)
Social capital	Can expect to help maximum 2 people in case of personal problems	0.78 (0.50; 1.20)	0.90 (0.51; 1.60)
	Others do not show much interest to him/her	1.02 (0.64; 1.62)	1.08 (0.58; 2.01)
	Not easy to receive help from the neighbours if he/she would need it	1.38 (0.90; 2.11)	1.72 (0.98; 3.03)
	He/she cannot talk to anyone about his/her personal cases	0.99 (0.35; 2.83)	0.88 (0.19; 3.95)
Access to health care	Late medical care because of waiting	1.80 (0.74; 4.40)	1.95 (0.46; 7.80)
	Late medical care because of long distance	1.36 (0.39; 4.78)	3.11 (0.54; 17.85)
Access to preventive services	This year or last year got flu vaccine	2.88 (0.64; 12.91)	1.64 (0.19; 14.14)
	Cholesterol level was measured in the last year	0.75 (0.47; 1.18)	0.81 (0.44; 1.49)
	Blood glucose level was measured in the last year	0.76 (0.49; 1.18)	0.65 (0.36; 1.17)
	Mammography examination in the last 2 years	0.54 (0.26; 1.12)	0.88 (0.36; 2.17)
	Cytological examination in the last 3 years	0.54 (0.29; 1.01)	0.90 (0.40; 2.00)
Adherence in drug consumption	People who take no medicines in case of musculoskeletal disorders	0.83 (0.30; 2.31)	0.37 (0.03; 4.37)
	People who take no medicines in case of cardio-metabolic diseases	1.08 (0.30; 3.86)	1.14 (0.20; 6.48)
	People who take no medicines in case of digestive and excretory system diseases	0.27 (0.02; 4.27)	nc
	People who take no medicines in case of respiratory system disorders (allergic diseases also)	1.03 (0.23; 4.70)	nc
Oral health	Carious tooth/cavity	2.20 (1.42; 3.39) *	4.30 (2.36; 7.85) *
	Dental fillings	0.43 (0.28; 0.66) *	0.73 (0.40; 1.32)
	Bleeding gums when tooth brushing	1.55 (0.92; 2.60)	2.86 (1.55; 5.30) *
	Lost teeth	1.91 (1.08; 3.37) *	2.46 (1.17; 5.17) *
	Pulled out teeth because of dental caries or loose teeth	1.10 (0.69; 1.77)	2.51 (1.28; 4.91) *
	Prosthesis or other type of dentures	0.67 (0.38; 1.18)	0.63 (0.28; 1.44)
	Missing teeth without prosthesis	1.89 (1.20; 2.98) *	3.09 (1.63; 5.89) *
	No dental filling, but he/she has cavity	3.53 (1.97; 6.30) *	3.92 (1.86; 8.27) *

* Significant results.; nc: not computed.

## References

[1] (2011). Communication from the Commission to the European Parliament, the Council, the European Economic and Social Committee and the Committee of the Regions: An EU Framework for National Roma Integration Strategies up to 2020.

[2] Marmot M. (2013). Health Inequalities in the EU—Final Report of a Consortium.

[3] Di Iorio C.T., Carinci F., Oderkirk J. (2014). Health research and systems’ governance are at risk: Should the right to data protection override health?. J. Med. Ethics.

[4] (2017). Communication from the Commission to the European Parliament and the Council: Midterm Review of the EU Framework for National Roma Integration Strategies.

[5] Dimitrova R., Johnson D.J., van de Vijver F.J.R. (2018). Ethnic socialization, ethnic identity, life satisfaction and school achievement of Roma ethnic minority youth. J. Adolesc..

[6] Dzhambov A.M., Dimitrova D.D. (2016). Association between Noise Pollution and Prevalent Ischemic Heart Disease. Folia Med..

[7] Dimitrova R., Chasiotis A., Bender M., van de Vijver F.J. (2014). Collective identity and well-being of Bulgarian Roma adolescents and their mothers. J. Youth Adolesc..

[8] Latorre-Arteaga S., Gil-Gonzalez D., Vives-Cases C., La Parra Casado D. (2017). Vision and Hearing Health Inequities in the Roma population: A National Cross-Sectional Study in Spain. J. Immigr. Minor. Health.

[9] Alvaro J.L., Morais de Oliveira T., Torres A.R., Pereira C., Garrido A., Camino L. (2015). The Role of Values in Attitudes towards Violence: Discrimination against Moroccans and Romanian Gypsies in Spain. Span. J. Psychol..

[10] Rosicova K., Reijneveld S.A., Madarasova Geckova A., Stewart R.E., Rosic M., Groothoff J.W., van Dijk J.P. (2015). Inequalities in mortality by socioeconomic factors and Roma ethnicity in the two biggest cities in Slovakia: A multilevel analysis. Int. J. Equity Health.

[11] Bobakova D., Dankulincova Veselska Z., Babinska I., Klein D., Madarasova Geckova A., Cislakova L. (2015). Differences between Roma and non-Roma in how social support from family and friends helps to overcome health care accessibility problems. Int. J. Equity Health.

[12] Logar M., Pavlic D.R., Maksuti A. (2015). Standpoints of Roma women regarding reproductive health. BMC Women’s Health.

[13] Zelko E., Svab I., Maksuti A., Klemenc-Ketis Z. (2015). Attitudes of the Prekmurje Roma towards health and healthcare. Wien. Klin. Wochenschr..

[14] Stamenkovic Z., Djikanovic B., Laaser U., Bjegovic-Mikanovic V. (2016). The role of mother’s education in the nutritional status of children in Serbia. Public Health Nutr..

[15] Hanssens L.G., Devisch I., Lobbestael J., Cottenie B., Willems S. (2016). Accessible health care for Roma: A gypsy’s tale a qualitative in-depth study of access to health care for Roma in Ghent. Int. J. Equity Health.

[16] Takaoka K., Gourtsoyannis Y., Hart J.D., Armstrong M., Daniel A., Mewse E., Phillips D., Bailey R.L. (2016). Incidence rate and risk factors for giardiasis and strongyloidiasis in returning UK travellers. J. Travel Med..

[17] Condon L.J., Salmon D. (2015). ‘You likes your way, we got our own way’: Gypsies and Travellers’ views on infant feeding and health professional support. Health Expect. Int. J. Public Particip. Health Care Health Policy.

[18] Smith D., Ruston A. (2013). ‘If you feel that nobody wants you you’ll withdraw into your own’: Gypsies/Travellers, networks and healthcare utilisation. Sociol. Health Illn..

[19] Balazs P., Rakoczi I., Grenczer A., Foley K.L. (2013). Risk factors of preterm birth and low birth weight babies among Roma and non-Roma mothers: A population-based study. Eur. J. Public Health.

[20] Balazs P., Fogarasi-Grenczer A., Rakoczi I., Foley K.L. (2014). Birth weight of Roma neonates: Effect of biomedical and socioeconomic factors in Hungary. Orv. Hetil..

[21] Kosa Z., Moravcsik-Kornyicki A., Dioszegi J., Roberts B., Szabo Z., Sandor J., Adany R. (2015). Prevalence of metabolic syndrome among Roma: A comparative health examination survey in Hungary. Eur. J. Public Health.

[22] Balazs P., Rakoczi I., Grenczer A., Foley K.L. (2014). Birth-weight differences of Roma and non-Roma neonates—public health implications from a population-based study in Hungary. Cent. Eur. J. Public Health.

[23] Szalai R., Matyas P., Varszegi D., Melegh M., Magyari L., Jaromi L., Sumegi K., Duga B., Kovesdi E., Hadzsiev K. (2014). Admixture of beneficial and unfavourable variants of GLCCI1 and FCER2 in Roma samples can implicate different clinical response to corticosteroids. Mol. Biol. Rep..

[24] Weber A., Szalai R., Sipeky C., Magyari L., Melegh M., Jaromi L., Matyas P., Duga B., Kovesdi E., Hadzsiev K. (2015). Increased prevalence of functional minor allele variants of drug metabolizing CYP2B6 and CYP2D6 genes in Roma population samples. Pharmacol. Rep. PR.

[25] Nagy A., Szalai R., Magyari L., Bene J., Toth K., Melegh B. (2015). Extreme differences in SLCO1B3 functional polymorphisms in Roma and Hungarian populations. Environ. Toxicol. Pharmacol..

[26] Szalai R., Ganczer A., Magyari L., Matyas P., Bene J., Melegh B. (2015). Interethnic differences of cytochrome P450 gene polymorphisms may influence outcome of taxane therapy in Roma and Hungarian populations. Drug Metab. Pharmacokinet..

[27] Kosa K., Adany R. (2007). Studying vulnerable populations: Lessons from the Roma minority. Epidemiology.

[28] Martinez-Cruz B., Mendizabal I., Harmant C., de Pablo R., Ioana M., Angelicheva D., Kouvatsi A., Makukh H., Netea M.G., Pamjav H. (2016). Origins, admixture and founder lineages in European Roma. Eur. J. Hum. Genet..

[29] Arora V.S., Kuhlbrandt C., McKee M. (2016). An examination of unmet health needs as perceived by Roma in Central and Eastern Europe. Eur. J. Public Health.

[30] Morar B., Gresham D., Angelicheva D., Tournev I., Gooding R., Guergueltcheva V., Schmidt C., Abicht A., Lochmuller H., Tordai A. (2004). Mutation history of the Roma/Gypsies. Am. J. Hum. Genet..

[31] Kuhlbrandt C., Footman K., Rechel B., McKee M. (2014). An examination of Roma health insurance status in Central and Eastern Europe. Eur. J. Public Health.

[32] Berg A.O., Andreassen O.A., Aminoff S.R., Romm K.L., Hauff E., Melle I. (2014). The impact of immigration and visible minority status on psychosis symptom profile. Soc. Psychiatry Psychiatr. Epidemiol..

[33] Schiek D., Lawson A. (2011). European Union Non-Discrimination Law and Intersectionality: Investigating the Triangle of Racial, Gender and Disability Discrimination.

[34] Dar O., Gobin M., Hogarth S., Lane C., Ramsay M. (2013). Mapping the Gypsy Traveller community in England: What we know about their health service provision and childhood immunization uptake. J. Public Health.

[35] Segregur J., Segregur D. (2017). Antenatal characteristics of Roma female population in Virovitica-Podravina County, Croatia. Zdr. Varst..

[36] Gresham D., Morar B., Underhill P.A., Passarino G., Lin A.A., Wise C., Angelicheva D., Calafell F., Oefner P.J., Shen P. (2001). Origins and divergence of the Roma (Gypsies). Am. J. Hum. Genet..

[37] Makowka A., Paradowska-Stankiewicz I., Szenborn L., Santibanez S., Mankerz A., Litwinska B. (2014). Measles outbreak among Roma people in Wroclaw, Poland, 2012. Pol. J. Microbiol..

[38] Hotchkiss D.R., Godha D., Gage A.J., Cappa C. (2016). Risk factors associated with the practice of child marriage among Roma girls in Serbia. BMC Int. Health Hum. Rights.

[39] Nikolaidis C., Nena E., Agorastakis M., Constantinidis T.C. (2016). Differences in survival and cause-specific mortality in a culturally diverse Greek population, 1999–2008. J. Public Health.

[40] Hubkova B., Maslankova J., Stupak M., Guzy J., Kovacova A., Pella D., Jarcuska P., Marekova M. (2014). Assessment of clinical biochemical parameters in Roma minority residing in eastern Slovakia compared with the majority population. Cent. Eur. J. Public Health.

[41] Kaditis A.G., Gourgoulianis K., Tsoutsou P., Papaioannou A.I., Fotiadou A., Messini C., Samaras K., Piperi M., Gissaki D., Zintzaras E. (2008). Spirometric values in Gypsy (Roma) children. Respir. Med..

[42] Tothova V., Bartlova S., Sedova L., Olisarova V., Prokesova R., Adamkova V., Mauritzova I., Treslova M., Chloubova I., Miksova Z. (2015). The importance of self-management in the prevention and treatment of excessive weight and obesity. Neuro Endocrinol. Lett..

[43] Adamkova V., Hubacek J.A., Novakova D., Dolak F., Adamek V., Lanska V., Tothova V., Sedova L. (2015). Genetic and biochemical characteristics in the Roma minority in the South Bohemia Region. Neuro Endocrinol. Lett..

[44] Sedova L., Tothova V., Olisarova V., Adamkova V., Bartlova S., Dolak F., Kajanova A., Mauritzova I., Novakova D., Prokesova R. (2015). Evaluation of selected indicators of overweight and obesity of Roma minority in the region of South Bohemia. Neuro Endocrinol. Lett..

[45] Antolova D., Jarcuska P., Janicko M. (2015). Seroprevalence of human Toxocara infections in the Roma and non-Roma populations of Eastern Slovakia: A cross-sectional study. Epidemiol. Infect..

[46] Poveda A., Ibanez M.E., Rebato E. (2014). Common variants in BDNF, FAIM2, FTO, MC4R, NEGR1, and SH2B1 show association with obesity-related variables in Spanish Roma population. Am. J. Hum. Biol..

[47] Brcanski J., Jovic-Vranes A., Marinkovic J., Favre D. (2014). Social determinants of malnutrition among Serbian children aged <5 years: Ethnic and regional disparities. Int. J. Public Health.

[48] Basic-Jukic N., Novosel D., Juric I., Kes P. (2013). Renal transplantation in the Roma ethnicity-do all patients have equal chance for transplantation?. Transplant. Proc..

[49] Ekmekci P.E. (2016). Health and Roma People in Turkey. Balk. Med. J..

[50] Beljic Zivkovic T., Marjanovic M., Prgomelja S., Soldatovic I., Koprivica B., Ackovic D., Zivkovic R. (2010). Screening for diabetes among Roma people living in Serbia. Croat. Med. J..

[51] Petek D., Rotar Pavlic D., Svab I., Lolic D. (2006). Attitudes of Roma toward smoking: Qualitative study in Slovenia. Croat. Med. J..

[52] Janevic T., Sripad P., Bradley E., Dimitrievska V. (2011). “There’s no kind of respect here” A qualitative study of racism and access to maternal health care among Romani women in the Balkans. Int. J. Equity Health.

[53] Hassler S., Eklund L. (2012). Sense of coherence and self-reported health among Roma people in Sweden—A pilot study. Int. J. Circumpolar Health.

[54] Martin-Perez M., Hernandez Barrera V., Lopez de Andres A., Jimenez-Trujillo I., Jimenez-Garcia R., Carrasco-Garrido P. (2015). Predictors of medication use in the Roma population in Spain: A population-based national study. Public Health.

[55] Chamova T., Guergueltcheva V., Gospodinova M., Krause S., Cirak S., Kaprelyan A., Angelova L., Mihaylova V., Bichev S., Chandler D. (2015). GNE myopathy in Roma patients homozygous for the p.I618T founder mutation. Neuromuscul. Disord. NMD.

[56] Peinado-Gorlat P., Castro-Martinez F.J., Arriba-Marcos B., Melguizo-Jimenez M., Barrio-Cantalejo I. (2015). Roma Women’s Perspectives on End-of-Life Decisions. J. Bioeth. Inq..

[57] Halanova M., Jarcuska P., Kalinova Z., Carikova K., Oravcova J., Jarcuska P., Pella D., Marekova M., Geckova A.M., Cislakova L. (2014). The prevalence of Chlamydia trachomatis in the population living in Roma settlements: A comparison with the majority population. Cent. Eur. J. Public Health.

[58] Gouva M., Mentis M., Kotrotsiou S., Paralikas T., Kotrotsiou E. (2015). Shame and Anxiety Feelings of a Roma Population in Greece. J. Immigr. Minor. Health.

[59] Stankovic S., Zivic S., Ignjatovic A., Stojanovic M., Bogdanovic D., Novak S., Vucic J., Stankovic M., Saranac L., Vesna C. (2016). Comparison of weight and length at birth of non-Roma and Roma newborn in Serbia. Int. J. Public Health.

[60] Lee E.J., Keyes K., Bitfoi A., Mihova Z., Pez O., Yoon E., Masfety V.K. (2014). Mental health disparities between Roma and non-Roma children in Romania and Bulgaria. BMC Psychiatry.

[61] Sudzinova A., Nagyova I., Rosenberger J., Studencan M., Vargova H., Middel B., van Dijk J.P., Reijneveld S.A. (2015). Seven years’ mortality in Roma and non-Roma patients after coronary angiography. Eur. J. Public Health.

[62] Kolvek G., Podracka L., Rosenberger J., Stewart R.E., van Dijk J.P., Reijneveld S.A. (2014). Kidney diseases in Roma and non-Roma children from eastern Slovakia: Are Roma children more at risk?. Int. J. Public Health.

[63] Sudzinova A., Rosenberger J., Stewart R.E., van Dijk J.P., Reijneveld S.A. (2016). Does poorer self-rated health mediate the effect of Roma ethnicity on mortality in patients with coronary artery disease after coronaro-angiography?. Int. J. Public Health.

[64] Sudzinova A., Nagyova I., Studencan M., Rosenberger J., Skodova Z., Vargova H., Middel B., Reijneveld S.A., van Dijk J.P. (2013). Roma coronary heart disease patients have more medical risk factors and greater severity of coronary heart disease than non-Roma. Int. J. Public Health.

[65] Masseria C., Mladovsky P., Hernandez-Quevedo C. (2010). The socio-economic determinants of the health status of Roma in comparison with non-Roma in Bulgaria, Hungary and Romania. Eur. J. Public Health.

[66] Gadalean F., Lighezan D., Stoian D., Schiller O., Timar R., Timar B., Bob F., Donciu M.D., Munteanu M., Mihaescu A. (2016). The Survival of Roma Minority Patients on Chronic Hemodialysis Therapy—A Romanian Multicenter Survey. PLoS ONE.

[67] Silarova B., van Dijk J.P., Nagyova I., Rosenberger J., Reijneveld S.A. (2014). Differences in health-related quality of life between Roma and non-Roma coronary heart disease patients: The role of hostility. Int. J. Public Health.

[68] (1995). Standards for the Classification of Federal Data on Race and Ethnicity.

[69] (2013). European Health Interview Survey (EHIS Wave 2) Methodological Manual.

[70] (2004). Human Development Report 2004.

[71] Kosa Z., Szeles G., Kardos L., Kosa K., Nemeth R., Orszagh S., Fesus G., McKee M., Adany R., Voko Z. (2007). A comparative health survey of the inhabitants of Roma settlements in Hungary. Am. J. Public Health.

[72] Nedo E., Paulik E. (2012). Association of smoking, physical activity, and dietary habits with socioeconomic variables: A cross-sectional study in adults on both sides of the Hungarian-Romanian border. BMC Public Health.

